# Hyperglycemia Potentiates Prothrombotic Effect of Aldosterone in a Rat Arterial Thrombosis Model

**DOI:** 10.3390/cells10020471

**Published:** 2021-02-22

**Authors:** Anna Gromotowicz-Poplawska, Piotr Szoka, Agnieszka Zakrzeska, Patrycjusz Kolodziejczyk, Natalia Marcinczyk, Janusz Szemraj, Piotr Tutka, Ewa Chabielska

**Affiliations:** 1 Department of Biopharmacy, Medical University of Bialystok, 15-222 Bialystok, Poland; natalia.marcinczyk@umb.edu.pl (N.M.); chabewa@poczta.onet.pl (E.C.); 2 Department of Pharmacology, Medical University of Bialystok, 15-222 Bialystok, Poland; szoka.piotr@gmail.com; 3 University of Medical Science of Bialystok, 15-875 Bialystok, Poland; a.zakrzeska@wsmed.pl; 4 Department of Experimental and Clinical Pharmacology, University of Rzeszow, 35-959 Rzeszow, Poland; patryk.kolodziejczyk@gmail.com (P.K.); tutka@umlub.pl (P.T.); 5 Department of Medical Biochemistry, Medical University of Lodz, 92-215 Lodz, Poland; janusz.szemraj@umed.lodz.pl; 6 National Drug and Alcohol Research Center, University of New South Wales, Sydney 2052, Australia

**Keywords:** aldosterone, adrenalectomy, diabetes, thrombosis, mineralocorticoid receptor, glucocorticoid receptor

## Abstract

We investigated the role of aldosterone (ALDO) in the development of arterial thrombosis in streptozotocin-induced diabetic rats. To evaluate the effect of endogenous ALDO, the rats underwent adrenalectomy (ADX). ADX reduced the development of arterial thrombosis. A 1 h infusion of ALDO (30 μg/kg/h) enhanced thrombosis in adrenalectomized rats, while this effect was potentiated in diabetic rats. ALDO shortened bleeding time, increased plasma levels of tissue factor (TF) and plasminogen activator inhibitor, decreased plasma level of nitric oxide (NO) metabolites, and increased oxidative stress. Moreover, 2 h incubation of human umbilical vein endothelial cells (HUVECs) with ALDO (10^−7^ M) disrupted hemostatic balance in endothelial cells in normoglycemia (glucose 5.5 mM), and this effect was more pronounced in hyperglycemia (glucose 30 mM). We demonstrated that the acute ALDO infusion enhances arterial thrombosis in rats and hyperglycemia potentiates this prothrombotic effect. The mechanism of ALDO action was partially mediated by mineralocorticoid (MR) and glucocorticoid (GR) receptors and related to impact of the hormone on primary hemostasis, TF-dependent coagulation cascade, fibrinolysis, NO bioavailability, and oxidative stress balance. Our in vitro study confirmed that ALDO induces prothrombotic phenotype in the endothelium, particularly under hyperglycemic conditions.

## 1. Introduction

There is growing evidence for a link between the activation of the renin–angiotensin–aldosterone system (RAAS) and cardiovascular-related complications such as unstable angina, myocardial infarction, and stroke [1,2]. The main pathological process responsible for these clinical events is acute intravascular arterial thrombosis or embolism. Aldosterone (ALDO), the final product of the RAAS system, plays a role in the pathogenesis of cardiovascular diseases in humans.

Primary aldosteronism (PA) is related to a higher percentage of strokes and myocardial infarctions, i.e., incidents associated with the arterial thrombotic process [3]. A high level of ALDO was found to be independently associated with cardiovascular mortality, total mortality, and acute ischemic events [1]. ALDO seems to influence the cardiovascular and hemostatic systems through several compound mechanisms, leading to increased risk of thrombosis. The proinflammatory and profibrotic effects of ALDO that cause structural, functional, and mechanical damage of vessels are attributed to ALDO-induced oxidative stress through modulation of NADPH oxidase [4]. In patients with PA, endothelium-dependent vasodilation was attenuated by the mechanism related to both increased NADPH oxidase and decreased eNOS expression [5]. A significant correlation was also observed between serum ALDO level and PAI-1 antigen [6]. Thus, ALDO has also been proposed to be a prothrombotic factor that was confirmed in animal models [7,8,9]. The acute administration of ALDO was demonstrated to enhance venous thrombosis in rats [9] and laser- or FeCl_3_-induced thrombosis in mesenteric venules in mice [10]. The compound mechanism of the prothrombotic action of ALDO is related to its rapid and simultaneous effects on platelets as well as plasma- and endothelium-dependent hemostatic factors and alteration in the clot structure, thereby making it resistant to fibrinolysis [9,10]. Moreover, it is known that vascular function that depends on smooth muscle cells and endothelium-dependent mechanisms affects the thrombotic process. ALDO has been shown to exert both deleterious and beneficial effects on the vasculature. It may promote vasodilation in situations with low levels of oxidative stress, while at higher oxidative stress levels followed by high salt intake, vascular injury, high oxygen tension, or inflammation, ALDO effect is associated with vasoconstriction [10,11,12,13]. All these data suggest that vascular health status, and the combination of endothelial dysfunction/damage and vascular oxidative stress are critical contributors to the net effect of ALDO in the vascular bed [14,15]. 

Oxidative stress, inflammation, and endothelial dysfunction are critical initiators for developing macro- and microvascular disorders, which are the principal cause of morbidity and mortality in patients with diabetes [16]. Hypercoagulable state and rheological abnormalities in diabetes contribute to acute and chronic thrombotic disorders [17,18]. There are considerable lines of evidence that ALDO contributes to the development of cardiovascular disorders in diabetes [19]. The involvement of ALDO in the process of arterial thrombosis in diabetes is, however, suggested only by few indirect clinical and experimental studies. It was observed that patients with diabetes with a small increase in plasma ALDO within the normal range showed a 10% increase in cardiovascular-related mortality [1]. Higher rates of cardiovascular events reported in PA could be partly due to the increased prevalence of metabolic syndrome in patients with this disorder [20]. A post hoc analysis of the EPHESUS trial demonstrated a greater total risk reduction in heart failure after myocardial infarction in diabetic patients than in nondiabetic individuals treated with eplerenone (EPL)—a selective mineralocorticoid receptor (MR) antagonist [21]. Moreover, in the preclinical studies, EPL decreased the dynamics of arterial thrombus formation and changed its structure in streptozotocin (STZ)-induced diabetic rats. The antithrombotic effect of EPL was related to reduced vessel wall hypertrophy, suppression of coagulation, inflammation and platelet activation, and fibrinolysis enhancement [22]. The mechanism of the antithrombotic action of EPL in experimental diabetes might also be related to improvement of NO bioavailability by reducing superoxide formation [23]. Our previous study showed improved endothelium-dependent vasorelaxation following EPL treatment in both large and small arteries of STZ-induced diabetic rats [24]. 

We previously observed that the effects of ALDO on hemostasis and venous thrombus formation were partially reduced after MR blockade with EPL and valsartan—an AT_1_ receptor antagonist [9,25]. The colocalization of MR and glucocorticoid receptor (GR) in the vascular endothelium and blood platelets suggests that GR receptors may also be responsible for the effect of ALDO on hemostasis. The effects of GR activation are closely related to endothelial dysfunction, inhibition of NO production, NADPH oxidase activation, increase in von Willebrand factor (vWF), and platelet activation [26,27,28]. It was also demonstrated that GR antagonist RU486 significantly suppressed PAI-1 induction in STZ-induced diabetic mice, indicating the involvement of GR in fibrinolysis impairment, thus thrombosis progression [29]. It is also important to know that the quantitative relationship between MR and GR occupancy by ALDO may depend on salt restriction or stress. Within the range of physiological levels of ALDO, its effects are predicted to be controlled by MR occupancy during circadian cycles and by MR and GR occupancy during salt restriction or acute stress [30]. Moreover, during the blockade of MR, ALDO as a nonselective agonist may bind to GR receptors [30,31]. Thus, the involvement of GR activation in the mechanisms of action of ALDO in hemostasis cannot be excluded.

Therefore, the present study was designed to evaluate the direct effects of endogenous and exogenous ALDO on arterial thrombosis development under hyperglycemic conditions in rats. To test this objective, we used adrenalectomized rats (ADX) as well as adrenalectomized rats with STZ-induced diabetes and acute ALDO infusion. We also investigated the role of MR and GR in ALDO-mediated prothrombotic action and their effects on hemostasis and oxidative stress in vivo. Moreover, an in vitro study was conducted to investigate the role of ALDO on hemostatic and oxidative status of the endothelium under normo- and hyperglycemic conditions. This is the first study to assess the direct role of ALDO in an in vivo model of arterial thrombosis development in diabetes. The results of the present study extend our previous findings regarding the involvement of ALDO in hemostasis regulation and demonstrate multiple mechanisms of ALDO action, thus showing the need of a search for new strategies of ALDO blockade in diabetes. 

## 2. Materials and Methods

### 2.1. Animals 

Male Wistar rats were used. All the animals were housed in group cages as appropriate in a room with a 12 h light/dark cycle and allowed access to tap water and standard rat/mouse food. Procedures involving the animals and their care were conducted according to the institutional guidelines that comply with the national and international laws, including EU Directive 2010/63/EU for animal experiments and the guidelines for the care and use of laboratory animals in biomedical research. All the procedures were approved by the Local Ethical Committee of Animal Testing at the Medical University of Bialystok (approval numbers: 13/2014, 54/2015, 55/2015).

### 2.2. Chemicals 

Aldosterone, amphotericin B, glucose, heparin, L-glutamine, mannitol, penicillin, RU486, streptokinase, streptomycin, streptozotocin, trypsin, and trypan blue were purchased from Sigma-Aldrich (Poznan, Poland). EPL (Inspra; Pfizer, Warsaw, Poland), low-serum growth supplement (LSGS; Cascade Biologics, Portland, OR, USA), Medium 200 (M200; Cascade Biologics, Portland, OR, USA), and pentobarbitone sodium (Morbital; Biowet, Pulawy, Poland) were used. EDTA, ethanol, gum arabic, natrium chloride, magnesium chloride, and trisodium citrate were purchased from Polish Chemical Reagents (Gliwice, Poland).

### 2.3. Bilateral Adrenalectomy

Rats were anesthetized with an injection of pentobarbitone sodium (50 mg/kg, *i.p.*). Adrenalectomy (ADX) was performed bilaterally through two dorsolateral midflank skin and muscular incisions. Sham surgeries (SHAM) were identical to adrenalectomies, except that the adrenals were not removed. Rats that underwent ADX were provided with 0.9% NaCl solution in their drinking water to maintain water and electrolyte balance [29]. Sham-operated control rats were given free access to standard chow and water ad libitum. 

### 2.4. STZ-Induced Diabetes 

Diabetes was induced with a single injection of STZ (65 mg/kg, *i.p.*) as previously described [22] two weeks after ADX. Diabetes developed in the animals in two weeks. Control rats were normoglycemic (NORM). Blood glucose level was monitored using a one-touch blood glucose meter (CardioCheck, Chorzow, Poland).

### 2.5. ALDO Infusion and Drug Administration

All investigations were performed at the same time of the day (9:00 a.m.) to minimize any effect of diurnal variation on hemostasis. ALDO (30 μg/kg/h) or vehicle (VEH, 0.04% ethanol; 2 mL/kg/h) was infused into the femoral vein of anesthetized rats 5 min before arterial thrombosis induction and was continued for 1 h. To assess the role of particular receptors, EPL (100 mg/kg, *p.o.*)—a selective MR antagonist, or RU486 (10 mg/kg, *s.c.*)—a GR antagonist, was administered 30 min before ALDO infusion. 

### 2.6. Arterial Thrombosis Induction

Arterial thrombosis was induced by an electrical stimulation of the right common carotid artery as previously described [32]. The artery stimulation (1 mA) took 10 min, and the subsequent thrombus progression led to a gradual reduction in carotid blood flow (CBF). CBF was monitored with a Doppler flow probe (HugoSachs Elektronik, March, Germany) placed in contact with the exposed artery, downstream of the electrode, and connected to a blood flowmeter (HugoSachs Elektronik, March, Germany). Total CBF was calculated by the trapezoidal rule that measures the area under the CBF-time curve with TP4.1 pharmacometric software (ThothPro^TM^, Gdansk, Poland) and normalized as percentage of baseline (0 min) flow over 55 min to provide a measure of average blood flow during thrombus formation [22,33]. The occlusion was defined as a lack of arterial blood flow (0 mL/min) maintained for 5 min. The total time to occlusion (TTO) was defined as the time from the start of the electrical stimulation until the occlusion. If the vessel did not occlude by 60 min, the TTO was assigned the 55 min value for data analysis. At the end of the experiment, thrombus was removed by the incision of the artery of anesthetized rat, air-dried, and weighed after 24 h. For ex vivo experiments, blood samples were collected from the right heart ventricle after thrombus removal, then rats were sacrificed.

### 2.7. Bleeding Time 

Bleeding time (BT) was recorded as described previously, prior to thrombus removal [32]. Briefly, a standardized device was applied longitudinally on the dorsal part of the tail between 6 and 9 cm from the tip, taking care to avoid the large veins. Immediately after injury, the tail was placed into a cylinder with isotonic saline at 37 °C, and bleeding time was measured from the moment the tail was surgically cut until bleeding completely stopped (no bleeding for < 30 s). 

### 2.8. ALDO and Corticosterone Level 

Corticosterone (CORT) plasma level was determined using the ELISA kit (DRG Instruments GmbH, Marburg, Germany). The ALDO serum level was determined by radioimmunoassay (Aldosterone CoatA-Count RIA Kit; Los Angeles, CA, USA).

### 2.9. Blood Morphology and Plasma Hemostatic Parameters

Blood morphology test was performed using a hematological analyzer (ScilVet ABC Plus+, HORIBA ABX, Grabels, France). Plasma levels of tissue factor (TF), tissue plasminogen activator (t-PA), and plasminogen activator inhibitor type 1 (PAI-1) were measured by enzyme immunoassays (Rabbit Monoclonal Antibody Anti Rat TF; ImmunoKontact AMS Biotechnology, Abingdon, UK; Rat Active tPA ELISA Kit, Innovative Research, Novi, MI, USA; Rat Active PAI-1 ELISA Kit; Innovative Research, Novi, MI, USA, respectively). 

### 2.10. Nitric Oxide Level

The plasma level of nitric oxide (NO) was measured colorimetrically as nitrite/nitrate (NO_2_/NO_3_) concentration with the Correlate Assay Nitric Oxide NO_2_/NO_3_ Assay Kit (Assay Designs, Ann Arbor, MI, USA). 

### 2.11. Oxidative Stress Parameters

The markers of oxidative stress, namely hydrogen peroxide (H_2_O_2_) and malonyl dialdehyde (MDA) levels, were assayed in plasma by using commercially available kits (Hydrogen Peroxide Colorimetric Detection Kit; Assay Designs and MDA Adducts ELISA Kit; Cell Biolabs, San Diego, CA, USA, respectively). 

### 2.12. Hemostasis, NO, and Oxidative Stress Evaluation in HUVECs

Cryopreserved human umbilical vein endothelial cells (HUVECs) were used (Cascade Biologics Inc., UK) for the in vitro study. The cells were grown in Medium 200 supplemented with penicillin/streptomycin (100 units/mL penicillin and 100 mg/mL streptomycin) and low-serum growth supplement at 37 °C in a 95% humidified atmosphere of 5% CO_2_. The medium was replaced every 2–3 days. At confluence, the cells were subcultured by trypsinization, following which the cells were seeded with a split ratio of 1:3. Cellular viability was determined by the trypan blue staining method. Cultures at 4–5 passages were used in the experiments. HUVECs were cultured for 24 h under two different conditions: normoglycemic—in a medium with 5.5 mM glucose and 24.5 mM mannitol, or hyperglycemic—in a medium with 30 mM glucose. After 24 h, the cells were incubated with VEH or ALDO (10^−7^ M). After 2 h of incubation, cell supernatants were collected, and TF, t-PA, PAI-I, NO_2_/NO_3_, H_2_O_2_, and MDA levels were measured with commercially available kits. Endothelial nitric oxide synthase (eNOS) expression at the mRNA level was determined by quantitative real-time PCR as previously described [9,34]. The following PCR primers were designed: 5′-CATCGGCGTGCTGCGGGATCAG-3′ and 5′-GGGCTGTTGGTGTCTGAGCCGG-3′, which were specific for the mRNAs of eNOS. The amount of eNOS mRNAs was quantified.

### 2.13. Statistical Analysis

The data are presented as mean ± SEM of the number of determinations (*n*). The Shapiro–Wilk test was performed to determine the normal distribution. Statistical analysis was performed using the nonparametric Mann–Whitney U test, since the results did not pass the normality test. Differences were considered significant at the *p*-value of <0.05. All analyses were performed in GraphPad Prism 5. 

## 3. Results

### 3.1. General Characteristic of Rats 

Plasma ALDO and CORT levels were below the limit of detection in adrenalectomized rats (ADX) vs. sham-operated (SHAM) rats. Compared to SHAM rats, ADX rats showed a marked decrease in hematocrit (*p* < 0.05), TF (*p* < 0.01), PAI-1 (*p* < 0.01), NO metabolites (*p* < 0.01), and MDA levels (*p* < 0.01), and an increase in t-PA (*p* < 0.01) and H*_2_*O*_2_* levels (*p* < 0.01). In STZ-induced diabetic rats (STZ), an increase in blood glucose (*p* < 0.01) and plasma ALDO levels (*p* < 0.05) was observed as compared to that in normoglycemic rats (NORM). An increase in the count of white blood cells (WBC; *p* < 0.05) and red blood cells (RBC; *p* < 0.01), and hemoglobin (HGB; *p* < 0.01) and hematocrit levels (HCT; *p* < 0.01) was also observed (vs. NORM). STZ rats showed a significant decrease in body weight (*p* < 0.01), platelet count (PLT; *p* < 0.05), and BT (*p* < 0.01) (vs. NORM). Plasma ALDO and CORT levels were undetectable in adrenalectomized STZ-induced diabetic rats (ADX STZ), although an increase in body weight (*p* < 0.05) and decrease in blood glucose level (*p* < 0.05) were observed (vs. STZ). ADX STZ rats also showed a decrease in RBC count (*p* < 0.05), HCT level (*p* < 0.05), and an increase in PLT count, BT (*p* < 0.05), and NO metabolite level (*p* < 0.05 vs. STZ) (Table 1).

### 3.2. Arterial Thrombosis in ADX, STZ, and ADX STZ Rats

**Figure 1:**
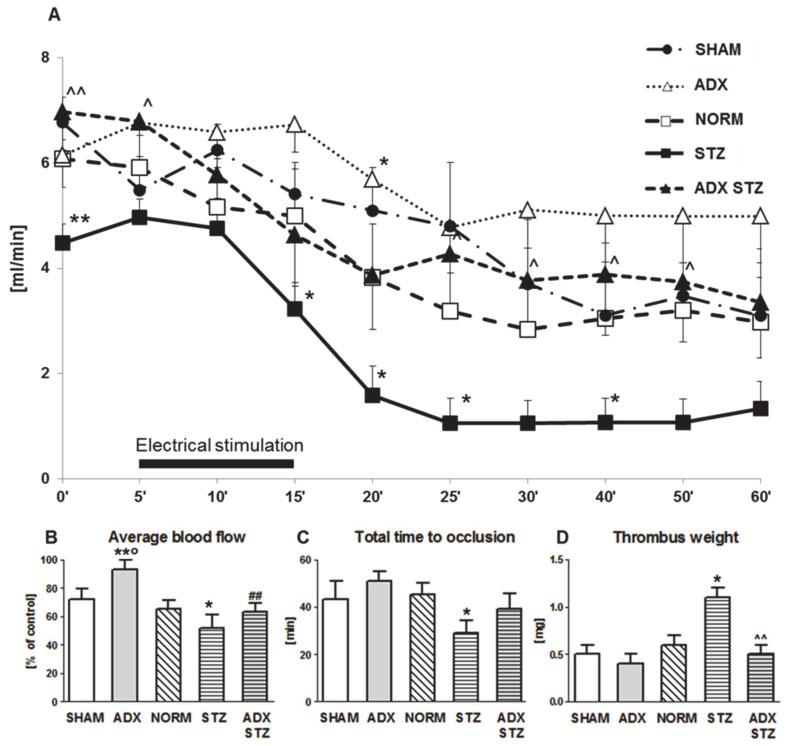
Time-course of mean blood flow and thrombus formation in a rat common carotid artery. (**A**) The mean carotid blood flow during the entire experiment; (**B**) average blood flow; (**C**) total time to occlusion; (**D**) thrombus weight; SHAM—sham-operated rats, ADX—adrenalectomized rats, NORM—normoglycemic, control rats, STZ—streptozotocin-induced diabetic rats, ADX STZ—adrenalectomized streptozotocin-induced diabetic rats. Data are presented as mean ± SEM; * *p* < 0.05, ** *p* < 0.01 vs. NORM; ° *p* < 0.05 vs. SHAM; ^ *p* < 0.05, ^^ *p* < 0.01 vs. STZ; ## *p* < 0.01 vs. ADX; *n* = 7–12.

In this set of in vivo experiments, the effect of exclusion of endogenous ALDO on the development of arterial thrombosis in NORM and STZ rats was determined. In ADX rats, an increase in the average CBF was observed as compared to that in SHAM rats (*p* < 0.05) (Figure 1B). Furthermore, compared to NORM rats, STZ rats showed increased prothrombotic potential expressed as decrease in the average CBF (*p* < 0.05; Figure 1B) and shortening of TTO (*p* < 0.05; Figure 1C). An approximately two-fold increase in thrombus weight was found in STZ rats as compared to that in NORM rats (*p* < 0.05; Figure 1D). There was no difference in the average CBF between STZ and ADX STZ rats, although a significant increase in the initial CBF was observed in ADX STZ rats (6.9 ± 0.7 vs. 4.5 ± 0.4; *p* < 0.01; Figure 1A). Compared to STZ rats, ADX STZ rats showed a significant reduction in thrombus weight (*p* < 0.01; Figure 1D).

### 3.3. The Effect of Acute ALDO Infusion on Arterial Thrombosis in ADX and ADX STZ Rats

**Figure 2:**
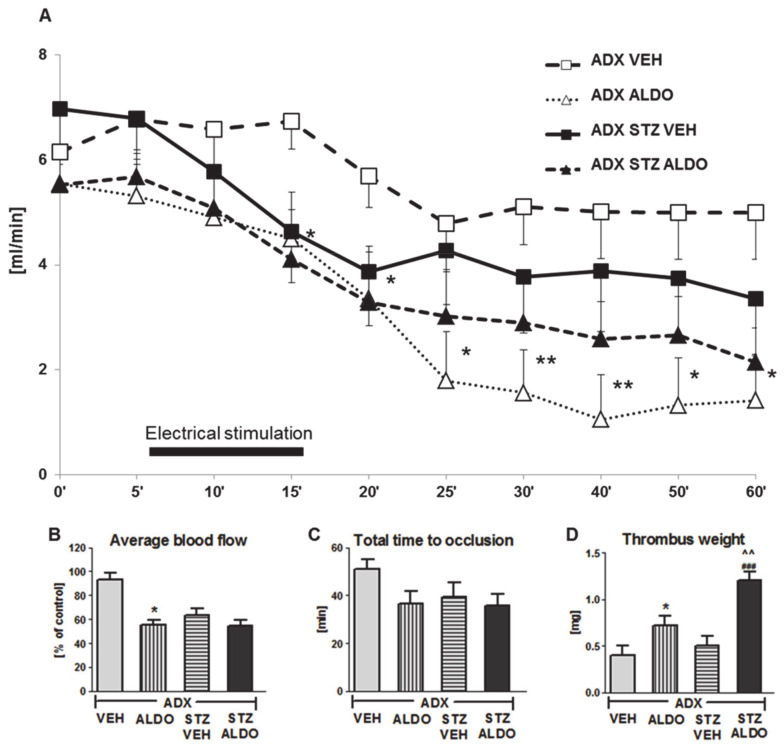
Time-course of mean blood flow and thrombus formation in common carotid artery during aldosterone infusion in adrenalectomized and adrenalectomized streptozotocin-induced diabetic rats. (**A**) The mean carotid blood flow during the entire experiment; (**B**) average blood flow; (**C**) total time to occlusion; (**D**) thrombus weight; ADX VEH—adrenalectomized rats, infused with vehicle; ADX ALDO—adrenalectomized rats, infused with aldosterone; ADX STZ VEH—adrenalectomized streptozotocin-induced diabetic rats, infused with vehicle; ADX STZ ALDO—adrenalectomized streptozotocin-induced diabetic rats, infused with aldosterone. Data are presented as mean ± SEM; * *p* < 0.05, ** *p* < 0.01 vs. ADX VEH; ### *p* < 0.001 vs. ADX STZ VEH; ^^ *p* < 0.01 vs. ADX ALDO; *n* = 7–12.

In the next set of in vivo experiments, the acute effect of 1 h ALDO infusion at the dose of 30 µg/kg on arterial thrombosis was determined. ALDO infusion resulted in an increase in plasma hormone level to 1950 ± 224 pg/mL in ADX STZ rats. The CBF monitored during the entire experiment is shown in Figure 2A. ALDO caused a significant reduction in the average CBF in ADX rats as compared to that in VEH-infused rats (*p* < 0.05; Figure 2B). ALDO also led to a marked increase in thrombus weight in both ADX ALDO (*p* < 0.05 vs. ADX VEH) and ADX STZ ALDO groups (*p* < 0.001 vs. ADX STZ VEH), while this effect was more pronounced in ADX STZ ALDO vs. ADX ALDO (*p* < 0.01; Figure 2D). 

Moreover, ALDO infusion in ADX STZ rats increased TF (*p* < 0.05), t-PA (*p* < 0.001), PAI-1 (*p* < 0.05), and H_2_O_2_ (*p* < 0.001) plasma levels, but decreased BT (*p* < 0.05) and reduced NO metabolite (*p* < 0.05) and MDA (*p* < 0.01) plasma levels (Table 2). 

### 3.4. The Role of MR and GR in the Prothrombotic Effect of ALDO in ADX STZ Rats 

**Figure 3:**
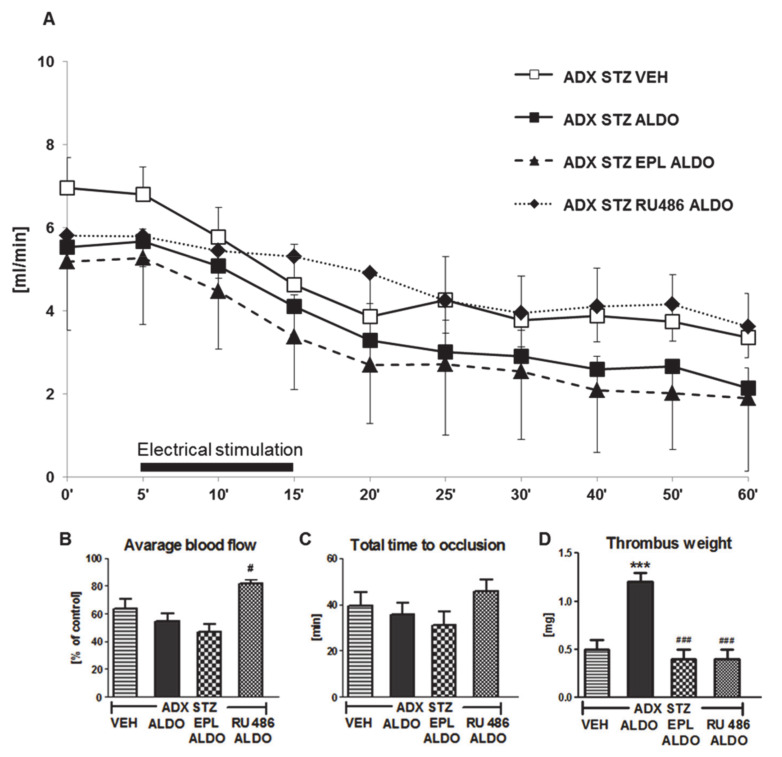
The role of mineralocorticoid and glucocorticoid receptors in the prothrombotic effect of aldosterone. (**A**) The mean carotid blood flow during the entire experiment; (**B**) average blood flow; (**C**) total time to occlusion; (**D**) thrombus weight; ADX STZ VEH—adrenalectomized streptozotocin-induced diabetic rats, infused with vehicle; ADX STZ ALDO—adrenalectomized streptozotocin-induced diabetic rats, infused with aldosterone; ADX STZ EPL ALDO—adrenalectomized streptozotocin-induced diabetic rats, administered with eplerenone and aldosterone; ADX STZ RU486 ALDO—adrenalectomized streptozotocin-induced diabetic rats, administered with RU486 and aldosterone. Data are presented as mean ± SEM; *** *p* < 0.001 vs. VEH; # *p* < 0.05, ### *p* < 0.001 vs. ALDO, *n* = 10–12.

EPL or RU486 coadministration with ALDO infusion caused a significant increase in ALDO plasma level (*p* < 0.01; Table 2). A marked increase in the average CBF was observed in ADX STZ RU486 ALDO (*p* < 0.05 vs. ADX STZ ALDO; Figure 3B). No significant differences in the TTO were observed in all groups (Figure 3C). Coadministration of EPL or RU486 significantly inhibited the ALDO-induced increase in thrombus weight (*p* < 0.001; Figure 3D). 

Coadministration of RU486 also caused significant prolongation of BT (*p* < 0.01, vs. ADX STZ ALDO). Both EPL and RU486 reduced TF plasma level in ADX STZ ALDO rats (*p* < 0.01). EPL-treated rats showed increase in t-PA and decrease in PAI-1 plasma levels (*p* < 0.001 and *p* < 0.01, respectively, vs. ADX STZ ALDO), while RU486 decreased only PAI-1 level (*p* < 0.01 vs. ADX STZ ALDO). EPL markedly increased the NO_2_/NO_3_ level as compared to that in the ADX STZ ALDO group (*p* < 0.001). Both EPL and RU486 reduced H_2_O_2_ and increased MDA plasma levels in ADX STZ ALDO rats (*p* < 0.001) (Table 2).

### 3.5. The Effect of ALDO on Hemostasis, NO Bioavailability, and Oxidative Stress in HUVECs under Hyperglycemic Conditions

HUVECs cultured in hyperglycemia (30 mM glucose) showed marked differences in hemostatic and oxidative stress parameters as compared to those cultured in normoglycemia (5.5 mM glucose). Hyperglycemia led to a significant increase in the level of TF (*p* < 0.01), PAI-1 (*p* < 0.001), H_2_O_2_ (*p* < 0.01), and MDA (*p* < 0.01), and a significant decrease in the level of t-PA (*p* < 0.01) and NO_2_/NO_3_ (*p* < 0.01) and the expression level of eNOS (*p* < 0.01). 

The results showed that preincubation of HUVECs in normoglycemic conditions with ALDO (10^−7^ M) disrupts hemostatic balance because of increased secretion of TF (*p* < 0.01) and PAI-1 (*p* < 0.01) and reduced release of t-PA (*p* < 0.01). Moreover, preincubation of HUVECs with ALDO reduced NO_2_/NO_3_ level (*p* < 0.01) and eNOS expression level, but it increased H_2_O_2_ (*p* < 0.001) and MDA levels (*p* < 0.05). 

The effects of ALDO on hemostasis, NO bioavailability, and oxidative stress in HUVECs were more pronounced under hyperglycemic conditions. ALDO showed more pronounced effects on TF (*p* < 0.01), t-PA (*p* < 0.05), PAI-1 (*p* < 0.01), NO_2_/NO_3_ (*p* < 0.01), eNOS expression (*p* < 0.01), H_2_O_2_ (*p* < 0.05), and MDA (*p* < 0.001) in hyperglycemia (Table 3). 

## 4. Discussion

In the present study, ADX and short-term ALDO infusion in adrenalectomized rats were used to elucidate the role of elevated ALDO level in mediating arterial thrombosis in diabetic rats. We used an STZ-induced diabetes mellitus model, a valid model of hyperglycemia that is associated with increased oxidative stress and vascular dysfunction [35]. The in vivo experiments were conducted in an electrically induced arterial thrombosis model that allows for continuous monitoring of the dynamics of thrombus formation and its lysis in the common carotid artery [36,37]. By using this model, we have previously established that arterial thrombus in STZ-induced diabetic rats showed an inflammatory nature expressed as an orderly arranged fibrous structure with densely packed platelets and numerous leukocytes as compared to that in normoglycemic animals. Alterations in plasma hemostatic parameters and in the systemic blood flow also occurred, which were observed as a significant reduction (>40%) in the initial CBF [22]. 

### 4.1. The Effect of ADX on Arterial Thrombosis

Our study showed that ADX decreased arterial thrombosis in normoglycemic rats. We observed a significant increase in the average blood flow in the carotid artery and a tendency to decrease in thrombus weight in adrenalectomized rats. On the basis of these findings, we conclude that the effect of ADX on arterial thrombosis in rats could be due to changes observed in primary hemostatic parameters and the effect on fibrinolysis activation and coagulation inhibition. Previously, it was reported that ADX increased t-PA activity in rat aorta [38]. It was also demonstrated that ADX prevented diabetes-induced increases in plasma PAI-1 levels and in PAI-1 mRNA expression in the heart and lungs in STZ-induced diabetic mice [29]. Moreover, some clinical data showed that ADX-induced normalization of ALDO secretion led to significant reduction of platelet activation expressed as reduced serum levels of soluble CD40L and *p*-selectin in patients with PA [39]. The antithrombotic effect of ADX, expressed as abrogated renal thrombotic microangiopathy, was shown previously in stroke-prone, spontaneously hypertensive rats (SHRSP). Protection provided by ADX in SHRSP rats was completely lost following two-week replacement with ALDO [7]. In the present study, ALDO infusion for even 1 h reduced the average blood flow and shortened TTO, leading to arterial thrombosis augmentation in adrenalectomized rats. The effect of ALDO in adrenalectomized rats may result from its antifibrinolytic and/or proinflammatory hemostatic effects [7,9,40]. The effect of ADX on thrombosis may also interfere with vascular NO bioavailability and oxidative stress balance, as we observed significant changes in oxidative stress parameters and NO metabolite level. It was shown that ADX reduced NADPH oxidase plasma levels and urinary isporostanes in patients with PA [41]. 

The regulation of MR expression and vascular density is suggested to be controlled by adrenal hormones [42]. Literature data on changes in MR expression after ADX are varied and depend on the phenotype of the cell. Increase or decrease in MR expression in various brain structures or the absence of changes in epithelial cells has been reported [43,44]; however, there are few data on MR expression in the cardiovascular system following ADX. There is evidence of an increase in heart ALDO levels because of ADX in SHRSP rats, while ALDO was not detected in plasma [45]. Our preliminary study showed 18% decrease in the aortic and 30% decrease in the heart MR protein level in adrenalectomized rats (data not shown). Further studies are required to determine whether the decrease in the local cardiovascular MR expression is a downregulation result of the local increase in ALDO levels. 

We cannot exclude that the effect of ADX on thrombosis could also be a result of exclusion of other adrenal hormones. It is known that catecholamines can alter vascular tone and platelet activation, the main elements involved in primary hemostasis and thrombus formation [46]. An intravenous injection of adrenaline stimulated arterial thrombosis formation in rats [47]. Experimental studies have suggested that glucocorticoids play a role in vascular and thrombotic disorders [48,49]. The procoagulant state and increased risk of thrombosis in patients with sustained exposure to glucocorticoids were also reported [50]. In summary, the effect of ADX on vascular and plasma hemostasis seems to be positive, which may be further confirmed by some clinical data [41,51].

### 4.2. Enhanced Arterial Thrombosis in STZ-Induced Diabetic Rats

Our findings indicate that thrombogenesis is more advanced in diabetic rats than in normal ones in the early stage of disease. We demonstrated for the first time the enhancement of experimental arterial thrombosis in the early (two-week) STZ-induced diabetic rats, observed as decrease in blood flow and dynamics of thrombus formation and its weight. We also observed significant changes in plasma and vascular hemostatic and oxidative stress parameters. Thus, we assume that the enhancement of arterial thrombosis in the early STZ-induced diabetic rats is related to vascular dysfunction, blood flow disturbances, and fibrinolysis impairment. Previously, it was demonstrated that platelets containing microthrombi accumulate in the retinal vasculature of the rat within two weeks of experimental STZ-induced diabetes [52]. Marked thrombogenesis was observed in the polyethylene tube-induced thrombosis in the carotid artery in three-week STZ-induced diabetic rats [53]. This effect was accompanied by a decrease in CBF. It was demonstrated that the decrease in CBF in STZ-induced diabetic rats is associated with vascular dysfunction due to upregulation of ET-1 biosynthesis in the vessel wall [54]. Endothelial injury expressed as an elevated plasma vWF level was observed previously in two-week STZ-induced diabetic rats [55]. 

We consider that our observations are important from the methodological point of view. The rat model of early STZ-induced diabetes can be used to evaluate the pathology of arterial thrombosis and activity of many biological substances and compounds for preventing thrombotic disorders in the early stage of diabetes. This model could also be used to evaluate the role of the ALDO/MR system in cardiovascular disorders in early diabetes, as we observed a significant increase in the ALDO plasma level in STZ-induced diabetic rats. 

### 4.3. Reduced Arterial Thrombosis in Adrenalectomized STZ-Induced Diabetic Rats

In the present study, bilateral ADX limited the development of arterial thrombosis in STZ-induced diabetic rats, as the increase in CBF and TTO and marked decrease in thrombus weight were observed. Although there were no changes in plasma hemostatic parameters, a significant increase in plasma NO metabolite level with increased CBF may suggest improvement in vascular function, leading to reduced arterial thrombosis. These changes were observed with no detectable plasma ALDO level.

Adrenalectomized STZ-induced diabetic rats showed a significant decrease in blood glucose level. Previously, it was shown that ADX attenuated STZ-induced increase in blood glucose level in mice [31]. The decrease in blood glucose level in adrenalectomized STZ-induced diabetic rats is related to enhancement of insulin sensitivity [56]. It was also reported that ALDO administration elevated blood glucose level in mice and that this effect was associated with GR activation and promotion of hepatic gluconeogenesis [57]. Thus, we assume that the decrease in glucose level in adrenalectomized STZ-induced diabetic rats could also be related to the attenuation of ALDO-induced gluconeogenesis. 

In conclusion, the mechanism of reduced arterial thrombosis in adrenalectomized STZ-induced diabetic rats could be related to reduced plasma ALDO level, which is well known as a prothrombotic factor [7,8,9,10], and to reduced blood glucose level, as hyperglycemia has been shown to be a potent inducer of PAI-1 expression [58]. Thus, ADX in STZ-induced diabetic rats may prevent the synergistic prothrombotic effect of both ALDO and hyperglycemia. 

### 4.4. Acute ALDO Infusion Enhanced Arterial Thrombosis in Adrenalectomized STZ-Induced Diabetic Rats 

To confirm that ALDO exclusion is responsible for reduced thrombosis in adrenalectomized rats, we performed experiments with an acute ALDO infusion. Here, we used ALDO at the dose of 30 μg/kg, which was found to be a prothrombotic dose in our previous study [9,10,25]. In the present study, ALDO infusion increased the plasma hormone level to 1950 ± 224 pg/mL in adrenalectomized STZ-diabetic rats. The supraphysiological plasma ALDO levels (3 to 20 times the normal level) may be found during circadian rhythm, hypertension, or heart failure [59,60,61]. 

We observed that the acute ALDO infusion enhanced arterial thrombosis in adrenalectomized rats and that this effect was more pronounced in adrenalectomized STZ-induced diabetic rats. Thus, we confirm that ALDO deficiency was responsible for reduced thrombus formation in adrenalectomized rats; moreover, we consider that the prothrombotic effect of ALDO is potentiated by hyperglycemia. Thus, the procoagulation state occurring in the early stage of diabetes in our study could be a result of hyperglycemia, increased ALDO level, and/or potentiated effects of hormone in hyperglycemic conditions. Hyperglycemia has been demonstrated to potentiate hypertrophic ALDO effect in neonatal rat cardiomyocytes [62]. However, the interaction between ALDO and hyperglycemia for the thrombotic process is presented here for the first time. 

ALDO-enhanced arterial thrombosis in adrenalectomized STZ-induced diabetic rats may be due to a compound mechanism of hormone prothrombotic action. We suggest that the decrease in CBF observed in the present study could be due to both ALDO-induced vasoconstriction and accumulation of thrombotic material in the carotid artery. We have previously shown that ALDO-dependent reduced CBF in diabetic rats occurs due to endothelium dysfunction and disturbed vasomotor function [24]. The endothelial overexpression of MR protects against FeCl_3_-induced arterial thrombosis in mice [14]. On the other hand, it was shown that the effects of ALDO on vasculature could depend on endothelium condition because in endothelial-denuded vascular ring segments, vasodilatory ALDO response was attenuated [63]. Thus, it seems that the final vascular effect of ALDO, that is, vasodilatation or vasoconstriction, may depend on the vascular health status [15].

We noticed that ALDO-enhanced thrombus weight in adrenalectomized STZ-induced diabetic rats was not paralleled by significant changes in TTO and CBF. The formation of a thrombus is the result of a disturbance of the normal balance between thrombogenesis and thrombolysis, thus different vascular and hemostatic mechanism involved. As a result of this, it is not surprising that under control conditions, thrombus growth can progress longitudinally, which allows thrombus size and mass to increase before occlusion is achieved [64]. It was shown that hyperglycemia had no effects on the time to occlusion, while more rapid thrombus formation was observed in hyperglycemic mice at the same time [65]. It was also reported that increased thrombogenicity of the vascular wall and platelet activation are more crucial for fibrin-rich thrombus formation than blood flow reduction [66]. Therefore, thrombus weight does not always translate into an equivalent change in TTO or CBF.

We observed that the augmentation of thrombotic process by acute ALDO infusion was partially mediated by MR- and GR-dependent mechanisms. The MR blockade significantly reduced the thrombus weight, with only slight effect on the dynamics of thrombus formation. It is likely that ALDO, as a nonselective agonist at the supraphysiological level, could induce GR-dependent action [67]. In fact, we found that GR blockade reduced the prothrombotic effect of ALDO, because an increase in CBF and reduction in thrombus weight were observed.

### 4.5. Effect of ALDO on Hemostasis

We showed that acute ALDO infusion significantly shortened BT in adrenalectomied STZ-induced diabetic rats, which indicates primary hemostasis activation and enhanced interaction of platelets with subendothelial structures. We previously reported that acute ALDO infusion reduced BT and increased platelet adhesion in rat venous thrombosis, and increased platelet activation in FeCl_3_-induced thrombosis in mice, and that these effects were MR-dependent [9,10]. The effect of ALDO on primary hemostasis may result from the direct effect of hormone on platelets. The MR receptor is present on the human platelet membrane and occurs as heterodimers with GR receptor subunits, which supports the hypothesis that platelets respond to ALDO [26]. ALDO has dual, agonist-dependent action on in vitro human platelet aggregation. It can augment platelet aggregation induced by collagen and reduce aggregation induced by arachidonate. EPL reverses the action of ALDO on collagen-dependent process, but augments the antiplatelet effect of ALDO in response to arachidonic acid [68]. Our preliminary study confirmed the presence of MR protein in rat platelets [10] and showed that the amount of MR protein in platelets of STZ-induced diabetic rats was slightly higher than that in normoglycemic rats (data not shown). Our results indicate that in diabetic rats, the effect of ALDO on BT was mainly GR-dependent. The involvement of GR in the modulation of platelet function was also reported by Moraes et al. The authors showed that preincubation of platelets with a GR agonist can reduce their response to ADP. However, this effect was ligand-dependent and associated with its affinity to MR and GR [26]. ALDO in the supraphysiological level can bind to both MR and GR; therefore, we suggest that enhanced primary hemostasis in our study could be related to multiple mechanisms.

TF is a key initiator of thrombogenesis, and its increased expression correlates with increased risk of thrombosis [69]. We observed that ALDO-induced thrombosis involved TF-dependent coagulation cascade. This effect was MR- and GR-dependent, as EPL and RU486 significantly reduced ALDO-increased TF plasma levels. In our previous study, chronic EPL therapy was found to reduce diabetes-induced activation of the TF-dependent coagulation pathway, thus emphasizing the role of MR in that process [22]. Lagrande et al. showed that ALDO increased thrombin generation in human smooth muscle cells, while reducing it in aortic endothelial cells, through MR-dependent mechanisms [14]. In the present study, we confirmed the role of GR in the regulation of TF-dependent pathways. A previous in vitro study showed that treatment of HUVECs with dexamethasone resulted in an increased induction of TF and vWF [70].

Our results indicate that ALDO-induced enhancement of arterial thrombosis was also related to impairment of fibrinolysis, which was expressed as an increase in PAI-1 plasma level. This effect was attenuated after MR- and GR-blockade. ALDO-induced upregulation of PAI-1 expression was shown previously in cultured renal mesangial cells and rat cardiomyocytes [71,72]. Chronic EPL treatment also decreased myocardial and renal mRNA expression of PAI-1 in rats [40,73]. The participation of GR in PAI-1 regulation was previously demonstrated in STZ-induced diabetic mice where RU486 administration decreased PAI-1 plasma levels [29]. The increase in t-PA plasma level after ALDO infusion could be a compensatory mechanism of increased PAI-1 level. The outcome of fibrinolytic activity is determined by balance in the blood of t-PA and PAI-1 antigen. Disturbed fibrinolytic balance expressed by increased t-PA and PAI-1 antigen levels was described in a variety of diseases ongoing with prothrombotic phenotype, e.g., deep venous thrombosis, myocardial infarction, or diabetes [22,74,75]. The increased levels of t-PA in the presence of decreased fibrinolytic activity have been interpreted to reflect accumulation of t-PA/PAI-1 inactive complexes. The beneficial effect of EPL and RU486 on plasma fibrinolysis parameters shows that both MR and GR receptors are involved in ALDO-induced fibrinolysis impairment. 

### 4.6. Oxidative Stress and NO Bioavailability in the Prothrombotic Action of ALDO

The proposed mechanism of ALDO prothrombotic effect includes decreased NO bioavailability and increased oxidative stress. We observed that ALDO induced decrease in NO_2_/NO_3_ and H_2_O_2_ plasma levels through MR- and/or GR-dependent mechanisms. Thus, we suggest that the possible mechanisms of ALDO-augmented arterial thrombosis could be related to endothelial dysfunction, decreased NO bioavailability, and, therefore, enhanced primary hemostasis and reduced vasomotor function. A clinical report demonstrated that acute 4 h ALDO infusion resulted in endothelial, NO-dependent vasodilator dysfunction in volunteers [76]. It was also demonstrated that a 14 day ALDO infusion caused significant impairment of endothelium-dependent vasodilatation in mice and treatment with EPL reversed this effect [77]. We previously showed that acute ALDO infusion decreased aortic eNOS expression and plasma NO metabolite level in venous thrombosis in healthy rats, although the effect was not MR dependent [9]. On the other hand, MR blockade was shown to improve endothelium-dependent vasorelaxation that was associated with reduction in superoxide formation and increase in NO bioavailability in STZ-induced diabetic rats [10,23]. However, in clinical reports, spironolactone worsened endothelial dysfunction in subjects with type 2 diabetes, which was reflected as attenuation of forearm blood flow response to acetylcholine. These findings were, however, possibly due to the deterioration of glycemic control and increase in plasma angiotensin II that occurred following spironolactone treatment [78]. This confirms that endothelium health status and pathological context may affect different NO-dependent vascular effects of ALDO.

ALDO-induced changes in CBF could be directly related to the action of reactive oxygen species (ROS) in the vasculature. It is well known that H_2_O_2_, produced in the vascular cells mainly by NOX4 isoform of NADPH oxidase, plays a role in the vascular function regulation. H_2_O_2_ usually induces vasodilatation but can also induce vasoconstriction or a dual effect depending on the concentration [79]. Thus, we conclude that the vascular effects of ALDO are mediated by the ROS-dependent mechanisms in vessels.

However, we observed in our in vivo models that two oxidative stress parameters (H_2_O_2_ and MDA) did not follow the same model-dependent trends, although it was not confirmed in our in vitro experiment. The mechanism responsible for the redox balance in the vasculature in vivo is complex, and the concentration of particular oxidative stress parameters that are produced by different metabolic pathways may not follow the same trend. 

In the present study, an increase in NO plasma level observed after MR blockade did not improve blood flow, although we cannot exclude that increased NO bioavailability affected platelet adhesion leading to decreased thrombus formation. We also postulate that decrease in TF and PAI-1 plasma levels after MR blockade could be correlated with increased NO metabolite level. In our study, GR blockade did not affect NO metabolite level. In contrast, it was previously demonstrated that GR participates in the modulation of NO bioavailability. Chronic treatment with dexamethasone attenuated vasorelaxation in response to acetylcholine that was associated with reduction in NO_2_/NO_3_ serum level and decrease in aortic eNOS expression in rats. An in vitro study involving incubation of HUVECs and bovine aortic endothelial cells with dexamethasone showed substantial downregulation of eNOS mRNA and protein through a GR-dependent mechanism [48]. We assume that these discrepancies are related to endothelial condition in diabetes. Our results show that an increase in arterial blood flow after GR blockade was not NO-dependent but still associated with primary hemostasis and coagulation inhibition, and fibrinolysis activation. 

### 4.7. Deleterious Effects of ALDO on HUVECs under Hyperglycemic Conditions 

Diabetes is known to induce endothelial dysfunction through well-known mechanisms, finally leading to increase in oxidative stress, which may be responsible for the thrombogenic phenotype of the endothelium [80]. Considering that the endothelium plays an important role in the regulation of arterial thrombus formation, in the next set of experiments, the effect of ALDO on endothelium-dependent hemostasis, NO bioavailability, and oxidative stress was determined in HUVECs. To date, the effect of ALDO on hemostasis in HUVECs under hyperglycemia has not been evaluated. First, our study extended previous observations showing increased coagulation and decreased fibrinolysis parameters, and reduced NO bioavailability and increased oxidative stress in HUVEC cultures under hyperglycemic condition as compared to that in normoglycemia [81,82]. The present study is the first to compare the acute effects of ALDO on endothelial hemostatic status in normoglycemic and hyperglycemic conditions. Incubation of HUVECs with ALDO (10^−7^ M) for 2 h induced strong prothrombotic phenotype of endothelial cells in normoglycemia, which was more pronounced in hyperglycemia. Our findings imply that the increase in glucose level may have a determining effect on the degree of ALDO-induced prothrombotic phenotype of the endothelium. Changes in hemostatic parameters may also result from reduced NO bioavailability and increased oxidative stress. The incubation of HUVECs under hyperglycemic conditions with ALDO decreased the level of NO metabolites, which was related to a reduction in eNOS expression. It is well known that decreased NO synthesis affects TF and fibrinolysis regulation and contributes to the deterioration of regulation of blood vessel wall tone, thereby promoting thrombus formation [83]. Thus, our in vitro results may further confirm our in vivo observations of the effects of ALDO in adrenalectomized STZ-induced diabetic rats with arterial thrombosis. The extensive effects of ALDO on endothelium-dependent coagulation and fibrinolysis, augmented under hyperglycemia, lead to acute hemostatic imbalance that promotes thrombosis. In conclusion, the effect of ALDO on the endothelium is a complex process and potentiates its thrombogenic phenotype particularly under hyperglycemic condition. 

In summary, we demonstrated that ADX can limit arterial thrombus formation in both normoglycemic and STZ-induced diabetic rats. Second, we showed that acute ALDO infusion enhances arterial thrombosis in adrenalectomized rats and that hyperglycemia potentiates this effect. Third, we confirmed the role of both MR and GR receptors in the prothrombotic action of ALDO. Finally, we demonstrated that the mechanism of the prothrombotic action of ALDO is related to the activation of primary hemostasis and TF-dependent coagulation, and fibrinolysis impairment. We also showed that ALDO induces prothrombotic phenotype of the endothelium, particularly under hyperglycemic conditions. This is the first direct evidence of the prothrombotic effect of ALDO on arterial thrombosis in early diabetes and extends the findings of our previous studies using the model of venous thrombosis in normoglycemic rats and mice [9,10,25].

### 4.8. Perspectives

Our study is a major step forward in better understanding of the mechanisms involved in thrombotic complications occurring during increase of ALDO in early diabetes. These data support a novel mechanism for thrombosis in which ALDO acts synergistically with endothelial injury and vascular oxidative stress observed in diabetes to enhance prothrombotic effect. Our results suggest that MR antagonists can reduce the development of thrombotic complications in diabetes; however, these results could be insufficient considering the ability of ALDO to also activate GR. Thus, our results suggest that alternative therapeutic strategies for modulating ALDO action on the cardiovascular system must be considered. Advancement of knowledge on the role of the acute pathway in the prothrombotic effect of ALDO and the role of GR may be crucial in exploring new strategies for the treatment of patients with elevated risk of cardiovascular events or thrombotic disorders associated with ALDO.

## Figures and Tables

**Table 1:** General characteristic of sham-operated, adrenalectomized, normoglycemic, and diabetic rats.

	SHAM (*n* = 7)	ADX (*n* = 7)	NORM (*n* = 9)	STZ (*n* = 12)	ADX STZ (*n* = 10)
**Body weight** **[g]**	405 ± 12	404 ± 15	415 ± 7	308 ± 5 **	346 ± 20 ^#^
**Glucose level** **[mg/dl]**	74 ± 4	70 ± 5	74 ± 3	269 ± 43 **	163 ± 18 ^#^ ^
**Survival rate** **[%]**	100	58	100	83	56
**Aldosterone level [pg/mL]**	678 ± 40	*n*/d	429 ± 58	786 ± 110 *	*n*/d
**Corticosterone level [pg/mL]**	430 ± 46	*n*/d	388 ± 60	480 ± 100	*n*/d
**WBC** **[10^3^/mm^3^]**	3.6 ± 0.3	4.0 ± 0.3	3.7 ± 0.4	5.3 ± 0.7 *	5.8 ± 1.3
**RBC** **[10^6^/mm^3^]**	7.2 ± 0.2	6.9 ± 0.3	7.6 ± 0.2	9.4 ± 0.6 **	7.7 ± 0.4 ^
**PLT** **[10^3^/mm^3^]**	912 ± 36	1034 ± 45	881 ± 44	665 ± 102 *	939 ± 102 ^
**HGB** **[g/dl]**	14.6 ± 0.4	13.8 ± 0.3	15.2 ± 0.1	17.9 ± 0.9 **	14.7 ± 0.8 ^
**HCT** **[%]**	42.9 ± 1.3	38.6 ± 1.2 °	48.7 ± 1.3	52.9 ± 0.6 **	43.4 ± 2.7 ^
**BT** **[s]**	88 ± 4	100 ± 11	99 ± 4	75 ± 4 **	80 ± 4 ^#^
**TF** **[pg/mL]**	5.3 ± 0.1	4.2 ± 0.1 °°	5.4 ± 0.1	5.2 ± 0.1	5.3 ± 0.1
**t-PA** **[ng/mL]**	6.4 ± 0.1	8.7 ± 0.1 °°	7.1 ± 0.1	5.5 ± 0.2 *	5.7 ± 0.1
**PAI-1** **[ng/mL]**	5.5 ± 0.1	4.0 ± 0.1 °°	6.0 ± 0.1	7.0 ± 0.1 *	7.2 ± 0.2
**NO_2_/NO_3_** **[μmol/l]**	10.7 ± 0.1	7.4 ± 0.1 °°	9.1 ± 0.1	10.7 ± 0.2 *	11.4 ± 0.1 ^
**H_2_O_2_** **[ng/mL]**	86.0 ± 0.1	111.9 ± 3.1 °°	91.7 ± 1.3	76.1 ± 2.5 *	76.3 ± 1.3
**MDA** **[pmol/mg]**	0.25 ± 0.01	0.17 ± 0.01 °°	0.28 ± 0.01	0.36 ± 0.02 *	0.33 ± 0.01

NORM—normoglycemic rats, SHAM—sham-operated rats, ADX—adrenalectomized normoglycemic rats, STZ—streptozotocin-induced diabetic rats, ADX STZ—adrenalectomized streptozotocin-induced diabetic rats; WBC—white blood cells, RBC—red blood cells, PLT—platelets, HGB—hemoglobin, HCT—hematocrit; BT—bleeding time; TF—tissue factor; t-PA—tissue plasminogen activator; PAI-1—plasminogen activator inhibitor; NO_2_/NO_3_ —nitric oxide metabolites; H_2_O_2_—hydrogen peroxide; MDA—malonyl dialdehyde. Data are presented as mean ± SEM; * *p* < 0.05, ** *p* < 0.01 vs. NORM; ° *p* < 0.05, °° *p* < 0.01 vs. SHAM; ^ *p* < 0.05 vs. STZ; # *p* < 0.05 vs. ADX; *n*/d, not detectable; *n*—number of rats.

**Table 2:** The role of mineralocorticoid and glucocorticoid receptors in the impact of aldosterone on hemostasis, nitric oxide bioavailability, and oxidative stress in adrenalectomized diabetic rats.

	VEH (*n* = 10)	ALDO (*n* = 12)	EPL + ALDO (*n* = 10)	RU486 + ALDO (*n* = 10)
**Aldosterone level** **[pg/mL]**	*n*/d	1950 ± 224	4192 ± 88 **	4054 ± 105 **
**BT** **[s]**	80 ± 4	66 ± 5 *	67 ± 4	97 ± 5 ^##^
**TF** **[pg/mL]**	5.3 ± 0.1	5.5 ± 0.1 *	5.1 ± 0.1 ^##^	5.1 ± 0.1 ^##^
**t-PA** **[ng/mL]**	5.7 ± 0.1	6.6 ± 0.1 ***	7.4 ± 0.1 ^###^	6.7 ± 0.1
**PAI-1** **[ng/mL]**	6.4 ± 0.2	7.3 ± 0.1 *	6.0 ± 0.1 ^##^	5.5 ± 0.2 ^##^
**NO_2_/NO_3_** **[µmol/l]**	11.4 ± 0.1	10.4 ± 0.3 *	12.8 ± 0.1 ^###^	9.6 ± 0.1
**H_2_O_2_** **[ng/mL]**	76.3 ± 13	98 ± 2.4 ***	75.0 ± 1.1 ^###^	66.2 ± 2.7 ^###^
**MDA** **[pmol/mg]**	0.33 ± 0.01	0.29 ± 0.1 **	0.5 ± 0.02 ^###^	0.63 ± 0.01 ^###^

ALDO—aldosterone; EPL—eplerenone, a selective mineralocorticoid receptor antagonist, RU486—a selective glucocorticoid receptor antagonist; VEH—vehicle; BT—bleeding time; TF—tissue factor; t-PA—tissue plasminogen activator; PAI-1—plasminogen activator inhibitor; NO_2_/NO_3_—nitric oxide metabolites; H_2_O_2_—hydrogen peroxide; MDA—malonyl dialdehyde. Data are presented as mean ± SEM; * *p* < 0.05, ** *p* < 0.01, *** *p* < 0.001 vs. VEH; ## *p* < 0.01, ### *p* < 0.001 vs. ALDO; *n*—number of rats.

**Table 3:** The effect of aldosterone on hemostasis, nitric oxide bioavailability, and oxidative stress parameters in the human umbilical vein endothelial cells under normoglycemic and hyperglycemic conditions.

	TF [pg/mL]	t-PA [ng/mL]	PAI-1 [ng/mL]	NO_2_/NO_3_ [µmol/l]	eNOS [2^-∆∆ct^]	H_2_O_2_ [ng/mL]	MDA [pmol/mg]
**NORM**	4.03 ± 0.02	8.11 ± 0.04	3.76 ± 0.02	10.61 ± 0.14	0.904 ± 0.003	105.0 ± 0.6	0.320 ± 0.004
**NORM VEH**	4.09 ± 0.03	8.08 ± 0.03	3.80 ± 0.01	10.81 ± 0.02	0.896 ± 0.009	105.8 ± 0.3	0.322 ± 0.001
**NORM ALDO**	4.96 ± 0.02 ^^	5.83 ± 0.02 ^^	6.10 ± 0.04 ^^	9.41 ± 0.13 ^^	0.791 ± 0.002 ^^	143.4 ± 0.7 ^^^	0.366 ± 0.002 ^
**GLU**	5.17 ± 0.01 **	6.86 ± 0.02 **	5.40 ± 0.04 ***	8.57 ± 0.17 **	0.728 ± 0.004 **	121.7 ± 0.1 **	0.404 ± 0.004 **
**GLU VEH**	5.18 ± 0.06	6.84 ± 0.04	5.36 ± 0.04	8.47 ± 0.11	0.723 ± 0.004	121.4 ± 0.1	0.406 ± 0.007
**GLU ALDO**	5.88 ± 0.03 ^##^ °°	5.10 ± 0.02 ^##^ °	10.55 ± 0.1 ^##^ °°	7.18 ± 0.04 ^##^ °°	0.613 ± 0.003 ^##^ °°	149.6 ± 0.3 ^##^ °	0.527 ± 0.004 ^##^ °°°

NORM—normoglycemic conditions; NORM VEH—normoglycemic conditions, vehicle incubation; NORM ALDO—normoglycemic conditions, aldosterone incubation; GLU—hyperglycemic conditions; GLU VEH—hyperglycemic conditions, vehicle incubation; GLU ALDO—hyperglycemic conditions, aldosterone incubation; TF—tissue factor; t-PA—tissue plasminogen activator; PAI-1—plasminogen activator inhibitor; NO_2_/NO_3_—nitric oxide metabolites; eNOS—endothelial nitric oxide synthase; H_2_O_2_—hydrogen peroxide; MDA—malonyl dialdehyde. Data are presented as mean ± SEM; ** *p* < 0.05, *** *p* < 0.001 vs. NORM; ^ *p* < 0.05, ^^ *p* < 0.01, ^^^ *p* < 0.001 vs. NORM VEH; ## *p* < 0.01 vs. GLU VEH; ° *p* < 0.05, °° *p* < 0.01,°°° *p* < 0.001 vs. NORM ALDO; *n* = 6.

## Data Availability

The raw data supporting the findings of this manuscript will be provided by the authors at any time to the reviewers and thereafter to any researcher after publication in *Cells*.
